# Mitochondrial dysfunction induces NLRP3 inflammasome activation during cerebral ischemia/reperfusion injury

**DOI:** 10.1186/s12974-018-1282-6

**Published:** 2018-08-28

**Authors:** Zhe Gong, Jingrui Pan, Qingyu Shen, Mei Li, Ying Peng

**Affiliations:** 1 Department of Neurology, Sun Yat-sen Memorial Hospital, Sun Yat-sen University, Guangzhou, 510120 China; 2 Guangdong Provincial Key Laboratory of Malignant Tumour Epigenetics and Gene Regulation, Sun Yat-sen Memorial Hospital, Sun Yat-sen University, Guangzhou, China

**Keywords:** Stroke, NLRP3 inflammasome, Mitochondrial dysfunction, Microglia, Neuron

## Abstract

**Background:**

Nod-like receptor protein 3 (NLRP3) inflammasome is a crucial factor in mediating inflammatory responses after cerebral ischemia/reperfusion (I/R), but the cellular location of NLRP3 inflammasome in cerebral I/R has yet come to a conclusion, and there is still no specific evidence to state the relationship between mitochondria and the NLRP3 inflammasome in cerebral I/R.

**Methods:**

In the present study, we detected the cellular localization of NLRP3 inflammasomes in a transient middle cerebral artery occlusion (tMCAO) rat model and a transwell co-culture cell system under oxygen-glucose deprivation/reoxygenation (OGD/R) conditions. Then, we investigated the relationship between mitochondrial dysfunction and the activation of NLRP3 inflammasomes in different cell types after OGD/R and cerebral I/R injury.

**Results:**

Our results showed that NLRP3 inflammasomes were first activated in microglia soon after cerebral I/R injury onset and then were expressed in neurons and microvascular endothelial cells later, but they were mainly in neurons. Furthermore, mitochondrial dysfunction played an important role in activating NLRP3 inflammasomes in microglia after OGD/R, and mitochondrial protector could inhibit the activation of NLRP3 inflammasomes in cerebral I/R rats.

**Conclusion:**

Our findings may provide novel insights into the cell type-dependent activation of NLRP3 inflammasomes at different stages of cerebral I/R injury and the role of mitochondrial dysfunction in activating the NLRP3 inflammasome pathway.

**Electronic supplementary material:**

The online version of this article (10.1186/s12974-018-1282-6) contains supplementary material, which is available to authorized users.

## Background

Neuroinflammation is a crucial and complex pathophysiological process within the whole scheme of cerebral ischemia, spanning from early damage to post-ischemic tissue repair [1]. The exact molecular signaling pathways have not been fully clarified to date, leading to difficulty in clinical treatment. Recently, a novel inflammatory pathway, known as inflammasomes, was found in ischemic stroke, and several studies have highlighted that nod-like receptor protein 3 (NLRP3) inflammasomes may be crucial for mediating inflammatory responses and for inducing cellular damage and death after stroke [2–4]. NLRP3 inflammasomes are the most well-characterized members of the nod-like receptor family, which consists of NLRP3, apoptosis-associated speck-like protein containing a caspase activation recruitment domain (ASC) and precursor caspase-1 (pro-caspase1), and it plays great roles in ischemic stroke by triggering the release of IL-1β and IL-18 via caspase-1 activation [5, 6]. Subsequently, both IL-1β and IL-18 participate in the initiation and amplification of the inflammatory responses [7]. However, the specific cellular location and signaling pathway of NLRP3 inflammasomes in ischemic stroke remains unknown.

In addition, it has been reported that mitochondrial dysfunction activates NLRP3 inflammasomes in some inflammatory diseases, such as metabolic syndrome, diabetes, atherosclerosis, neurodegeneration, heart disease, and kidney disease [8]. However, there is still no specific evidence stating the relationship between mitochondria and NLRP3 inflammasomes in stroke.

In the present study, we aimed to clarify these questions, in the hope of revealing more details regarding neuroinflammation in mediating cerebral ischemia/reperfusion (I/R) injury in ischemic stroke.

## Methods

### Animals and substances

Healthy, male, 280–320 g Sprague-Dawley rats were obtained from the Laboratory Animal Center of Sun Yat-sen University, Guangzhou, China. The rats were housed in a temperature- (25 ± 2 °C) and humidity-controlled room. The animals were maintained under a 12:12-h light/dark cycle with free access to food and water.

Diazoxide, considered a highly selective mitochondrial ATP-sensitive potassium channel opener, was dissolved in sterile 0.1 M NaOH solution before being used. The concentration of diazoxide used in the cells was 100 μm, which was in accordance with a previous study reported [9].

### Transient middle cerebral artery occlusion (tMCAO) model and drug treatment

The intraluminal suture MCAO method was used to induce tMCAO, as we previously described, in order to stimulate I/R injury in rats [10]. In brief, a midline incision was made in the neck to expose the right external carotid artery after each rat was anesthetized with 10% chloral hydrate. Then, a monofilament (Beijing Cinontech Co., Ltd.; China; 2838-A4) was inserted into the internal carotid artery, past the external carotid artery, until a mild resistance was felt, indicating that the filament was properly lodged in the proximal segment of the anterior cerebral artery and, thus, was blocking the blood flow to the middle cerebral artery. The monofilament was left in place for 120 min and then was withdrawn to induce reperfusion. When the rat regained consciousness, a successful model showed left foreleg paralysis or a circular motion, and infarct tissue without cerebral hemorrhage was observed when the brain was removed. A sham-operation was performed as a control, which included the same procedures as the tMCAO mentioned above but without the insertion of the monofilament and following reperfusion.

Rats were randomly divided into six groups: the sham-operation group (sham), the sham + diazoxide group, the 6 h after reperfusion group (I/R 6 h), the I/R 6 h + diazoxide group, the 24 h after reperfusion group (I/R 24 h), and the I/R 24 h + diazoxide group. The diazoxide was treated at 10 mg/kg intraperitoneally after reperfusion immediately as the previous studies [11, 12]. The diazoxide was dissolved in sterile 0.1 M NaOH solution, and diluted with saline to the concentration of 0.02 N NaOH solution before being used. The other animal group received equal volume of 0.02 N NaOH solution.

### Immunofluorescence (IF)

**Fig. 1:**
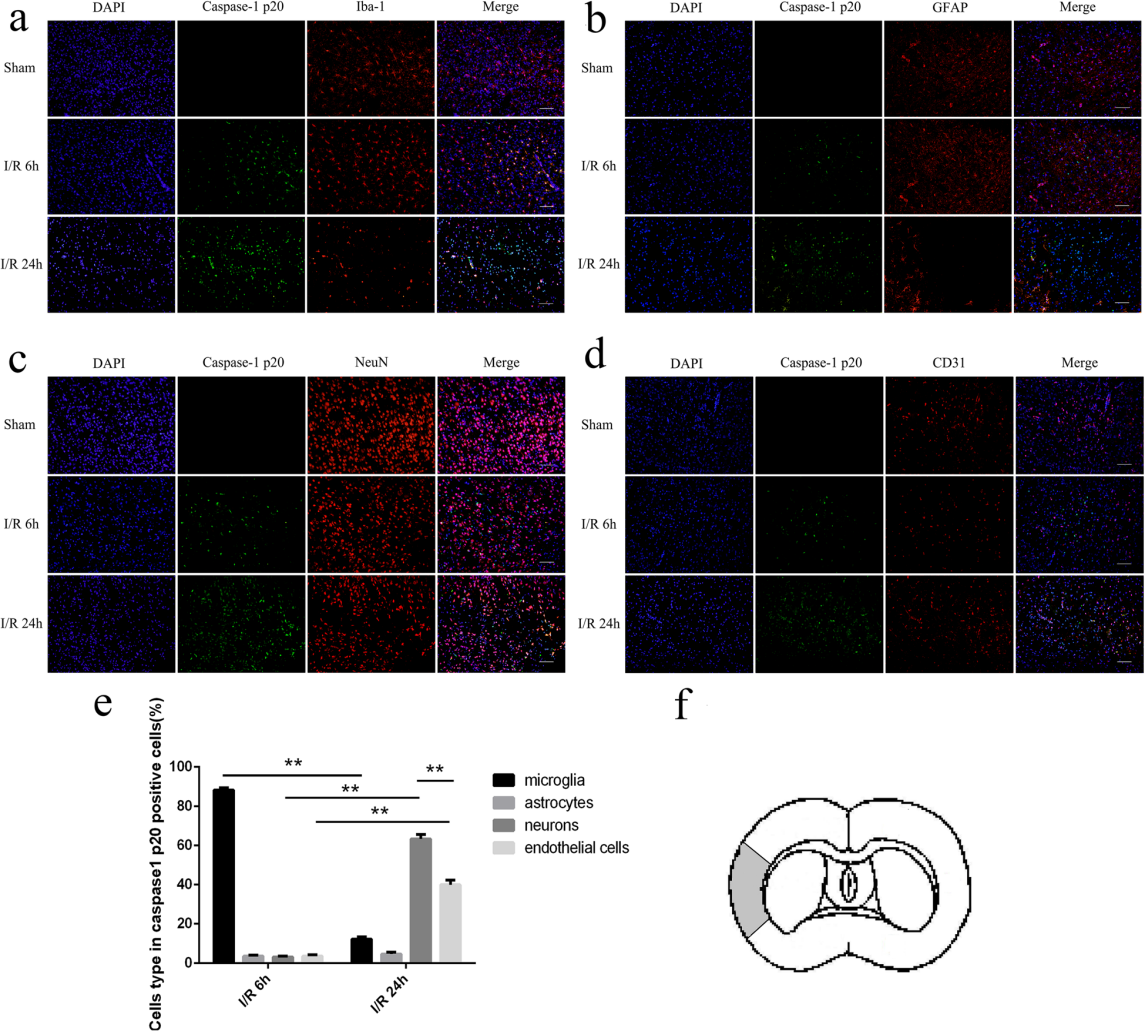
Cellular localization of cleaved caspase-1 after ischemia/reperfusion (I/R) injury. **a–d** The expression of caspase-1 p20 in microglia, astrocytes, neurons, and endothelial cells in sham rats and after cerebral I/R at 6 h and 24 h. **e** The percentage of different cell types in caspase-1 p20-positive cells 6 h and 24 h after cerebral I/R. **f** The ischemic area where the cleaved caspsae-1 was richly expressed. Bar = 100 μm. *p < 0.05, **p < 0.01

The brains were removed after cardiac perfusion 6 h and 24 h after reperfusion in different groups and were fixed with 4% paraformaldehyde at 4 °C overnight. Frozen sectioning was performed after sucrose gradient dehydration. Then, the 10-μm thick coronal sections were blocked with 10% goat serum containing 0.5% Triton X-100 at room temperature for 30 min, followed by incubation with mouse anti-caspase-1 p20 (Santa-Cruz Biotechnology, sc-398,715, 1:50) antibody and rabbit anti-Iba1 antibody (Wako, 019–19,741, 1:250) or rabbit anti-NeuN antibody (Cell Signaling Technology, 24307S, 1:50) or rabbit anti-CD31 antibody (Abcam, ab222783, 1:100) (The antibody specificity was proved in Additional file 1: Figure S1) or rabbit anti-GFAP antibody (Abcam, ab33922, 1:250) at 4 °C overnight. The sections were washed for 3 × 10 min with PBS and then incubated with a mixture of Alexa Fluor 488-conjugated goat-anti mouse IgG (Beyotime, A0428, 1:500) and Alexa Fluor 555-conjugated donkey-anti rabbit IgG (Beyotime, A0453, 1:500) for 1 h at room temperature, followed by staining with DAPI for 5 min. After being washed with PBS three times, the sections were mounted with fluorescent mounting medium (Dako, S3023). An equal volume of PBS was used to replace the primary antibody as a negative control, and the other procedures remained unchanged. All images that were focused on the ischemic core cortex area [13] (Fig. 1f) were captured using a fluorescence microscope (lx71, OLYMPUS) at × 200 magnification and a 100-μm scale bar. The percentage of different cell types in caspase-1 p20-positive cells is equal to the counts of both cell markers and caspase-1 p20-positive cells/counts of caspase-1 p20-positive cells. The percentage of caspase-1 p20-positive cells in different cell type is equal to the counts of both cell markers and caspase-1 p20-positive cells/counts of different cell type.

### Primary microglial cell culture

The method to isolate and cultivate primary microglial cells from C57BL/6 neonatal mice was according to a published protocol [14, 15]. Briefly, primary mixed glial cells were isolated from postnatal mice born within 24 h and then cultured in DMEM/F12 medium (Gibco, Invitrogen, 11,330–032) supplemented with 10% heat-inactivated fetal bovine serum (FBS) (Gibco, Invitrogen, 10,099–141) and 1% penicillin/streptomycin (HyClone, SV30010). On days 12–14, microglial cells were harvested by shaking the cultures and collecting the floating cells. After centrifugation, cells were seeded into plastic tissue culture flasks and incubated at 37 °C for 12–24 h, followed by culture medium replacement. The purity of microglia was verified by immunofluorescence staining with Iba-1 (Wako, 019–19,741, Japan).

BV2 cells, PC12 cells, and bEnd3 cells were obtained from American Type Culture Collection (ATCC). BV2 cells were cultured in DMEM/F12 medium (Gibco, Invitrogen, 11,330–032), and the other cells were cultured in DMEM high glucose (HyClone, SH30243.01), supplemented with 10% heat-inactivated fetal bovine serum (Gibco, Invitrogen, 10,099–141) and 1% penicillin/streptomycin (HyClone, SV30010) in a humidified incubator at 37 °C, in the presence of 5% CO_2_ in the air. For differentiation (Additional file 2: Figure S2), 100 ng/ml of nerve growth factor (NGF) (Sigma-Aldrich, N2513) was added to the culture medium of PC12 cells for 3 days, which was used for the following assays.

### Transwell co-culture system

The transwell co-culture system was conducted as previously reported [11]: BV2 cells were cultured on the upper compartment of a two-chamber transwell system (0.4-mm pore size of polycarbonate membrane coated with poly-L-lysine; Corning, Corning, NY, USA), and PC12 or bEnd3 cells were grown on the bottom well of the chamber, cultured in DMEM/F12 medium [16].

### siRNA transfection

Primary microglial cells, PC12 cells, bEnd3 cells, BV2 cells alone, and BV2 cells cultured in the upper chamber were transfected with NLRP3-siRNA (mouse) (forward, 5′-GUACUUAAAUCGUGAAACAdTdT-3′; reverse, 3′-dTdTCAUGAAUUUAGCACUUUGU-5′) or NLRP3-siRNA (rat) (forward, 5′-CAGCCAGAGUGGAAUG ACAdTdT-3′; reverse, 3′-dTdTGUCGGUCUCACCUUACUGU-5′) or NC-siRNA (RiboBio. CO., LTD, China) with Lipo3000 (Invitrogen, L3000–015) according to the manufacturer’s instructions. After 24 h of transfection, the treated upper chambers were moved to the other wells with PC12 or bEND3 cells cultured in the bottom chambers. Similarly, the treated PC12 or bEnd3 cells in bottom chambers were co-cultured with BV2 cells cultured in upper chambers. Then, the siRNA-transfected primary microglial cells, PC12 cells, bEnd3 cells, BV2 cells, and transwell co-culture systems received oxygen-glucose deprivation/reoxygenation (OGD/R) treatment.

The groups in transwell co-culture system, siRNA transfection, and OGD/R treatment were as following: Control treatment in isolated culture (NC), OGD/R treatment in isolated culture (OGD/R), OGD/R treatment in isolated culture with NC-siRNA transfection (OGD/R + NC siRNA), OGD/R treatment in isolated culture with NLRP3-siRNA transfection (OGD/R + NLRP3 siRNA), control treatment in transwell co-culture (transwell NC), OGD/R treatment in transwell co-culture (transwell OGD/R), OGD/R treatment in transwell co-culture with NC-siRNA transfection (transwell OGD/R + NC siRNA), and OGD/R treatment in transwell co-culture with NLRP3-siRNA transfection (transwell OGD/R + NLRP3 siRNA).

### Oxygen-glucose deprivation/reoxygenation (OGD/R) model and drug administration

The OGD/R model was generated by replacing the culture medium with glucose-free DMEM medium (Gibco, Invitrogen, 11,966–025), then placing the plate into a hypoxic incubator (MIC-101, Billups-Rothenberginc) that contained a gas mixture of 95% N_2_ and 5% CO_2_ for 4 h at 37 °C and then recovering normal gas and medium at the optimal time. The cell groups that needed drug treatment were treated with renewed normal medium with diazoxide after reoxygenation, and the other groups were treated with PBS as a control.

The groups in transwell co-culture system, drug treatment, and OGD/R treatment were as following: control treatment in isolated culture with PBS (NC), control treatment in isolated culture with diazoxide (NC + diazoxide), OGD/R treatment in isolated culture with PBS (OGD/R), OGD/R treatment in isolated culture with diazoxide (OGD/R + diazoxide), control treatment in transwell co-culture with PBS (transwell NC), OGD/R treatment in transwell co-culture with PBS (transwell OGD/R), and OGD/R treatment in transwell co-culture with diazoxide (transwell OGD/R + diazoxide).

### Flow cytometry

The apoptotic rates of the PC12 cells were detected using flow cytometry with an annexin V-FITC/PI apoptosis detection kit (KeyGEN BioTECH, KGA107) according to the manufacturer’s instructions. Briefly, isolated single-cell suspensions were stained with annexin-V and PI at room temperature in darkness for 15 min. Then, the number of cells was determined via flow cytometry (LSR II, BD).

### Measurement of mitochondrial DNA (mtDNA) copy number and mtDNA damage

Genomic DNA from the cells was extracted using the Genomic DNA Miniprep Kit (TIANGEN, DP304–02). The mtDNA copy number was measured using real-time quantitative PCR (qPCR) and was normalized to the Hbb (β-globin) gene. The primer pairs for measuring the mtDNA copy number were as follows: mtDNA forward, GCCCATGACCAACATAACTG; reverse, CCTTGACGGCTATGTTGATG; Hbb (β-globin) forward, AGGCAGAGGCAGGCAGAT; reverse, GG CGGGAGGTTTGAGACA. q-PCR reactions were performed in the LightCycler 480 II PCR System (LightCycler 480 II, Roche), using the All-in-One qPCR Mix kit (GeneCopoeia, AOPR-0200).

### Measurement of the mitochondrial membrane potential (∆ψm)

The ∆ψm was assayed using a JC-1 (5, 5′, 6, 6′-tetrachloro-1, 1′, 3, 3′-tetraethylbenzimidazolcarbocyanine iodide) staining Kit (Beyotime, C2006). Cells were incubated with 10 μg/ml JC-1 for 30 min at 37 °C, and images were captured with a fluorescence microscope. The ratio of JC-1 aggregates (red fluorescence) to monomers (green fluorescence) was calculated using Image-Pro Plus 6.0 (Media Cybernetics, lnc., USA). Loss of mitochondrial function by mPTP opening was indicated by a decrease in the ratio of the red/green fluorescence intensity [17].

### RNA extraction and real-time quantitative PCR (qRT-PCR)

The PC12 or bEnd3 cells were prepared for total RNA extraction using TRIzol reagent (Takara, #9109), and cDNA was synthesized using the PrimeScript RT Reagent Kit (Takara, #RR037A) according to the manufacturer’s instructions. The q-PCR reaction was performed in the LightCycler 480 II PCR System (LightCycler 480II, Roche, USA), using the All-in-One qPCR Mix kit (GeneCopoeia, AOPR-0200). Primers were provided as follows: IL-1β forward, TGCCACCTTTTGACAGTGATG and reverse, AAGGTCCACGGGAAAGACAC; IL-18 forward, AGCAGTCCCAACTAAGCAGTA and reverse CAGCCAGTAGAGGATGCTGA; and β-actin forward GTGACGTTGACATCCGTAAAGA and reverse GCCGGACTCATCGTACTCC. The endpoint of qRT-PCR data is the comparative cycle threshold method (Ct). The relative changes in gene expression were quantified using the Livak method (also known as 2^−ΔΔCt^ method) after determining the Ct values for the reference and target genes in each sample set [18]. All reactions were repeated for three times.

### Immunoprecipitation (IP)

The BV2 cell lysates (500 μg) were immunoprecipitated with 1 μg of anti-ASC antibody (Cell Signaling Technology, 67824S) for 1 h at 4 °C, and then were incubated with 20 μl of protein A agarose beads (Santa-Cruz, sc-2003) overnight at 4 °C, and centrifuged at 3000×*g* for 5 min. Protein complexes were washed five times with RIPA buffer, resuspended in × 2 loading buffer, and heated at 95 °C for 5 min. Then, the protein lysis buffers were used for western blot analysis with the following antibodies: rabbit anti-ASC (Cell Signaling Technology, 67824S, 1:1000), rabbit anti-NLRP3 (Cell Signaling Technology, #8242S, 1:1000), and mouse anti-caspase-1 (Santa-Cruz Biotechnology, sc-398,715, 1:100). Homophytic IgG was used as the negative control. SDS-PAGE and Western blot were used to analysis IP assay. The ASC protein was used as a loading control, and the loading quantities of precipitated materials were regulated according to the gray levels of ASC protein, to ensure brightness of reference bands were consistent. As the molecular weight of pro-caspase-1 and ASC were close to 50 kD or 25 kD, to avoid the influence of IgG light or heavy chain, the second antibodies used for pro-caspase-1 and ASC were anti-Mouse IgG Light Chain (Abbkine, A25012) and anti-Rabbit IgG Heavy Chain (Abbkine, A25222), respectively.

### Western blotting

Western blotting was performed according to conventional protocols. Briefly, the ischemic cortex or the cells were prepared for protein lysates using total protein lysis buffer (Beyotime, P0013) or IP protein lysis buffer (Beyotime, P0027) and were analyzed using SDS-PAGE (12%). The membranes were incubated with primary antibodies against NLRP3 (Cell Signaling Technology, #8242S, 1:1000), ASC (Cell Signaling Technology, 67824S, 1:1000), caspase-1 (Santa-Cruz Biotechnology, sc-398,715, 1:100), IL-1β (Santa-Cruz Biotechnology, sc-7887, 1:100), IL-18 (Abcam, ab71495, 1:125), and GADPH (Cell Signaling Technology, #2118S, 1:1000) at 4 °C overnight, followed by incubation with anti-rabbit IgG (MultiSciences (LiankeBio), GAR007, 1:5000), or anti-mouse IgG (MultiSciences (LiankeBio), GAM007, 1:5000) for 1 h at room temperature. The epitopes were visualized using an ECL western blot detection kit (KeyGEN BioTECH, KGP1126).

### Elisa

The supernatants were centrifuged and collected for ELISAs. The levels of the pro-inflammatory cytokines IL-1β (R&D Systems, MLB00C) and IL-18 (eBioscience, BMS618–3) were measured after OGD/R using commercial ELISA kits from eBioscience Systems. The procedures were performed according to the manufacturer’s instructions using a microplate reader (Bio-Rad, CA, USA).

### Statistical analysis

The Image-Pro Plus 6.0 (Media Cybernetics, lnc., USA) software was used to analyze the optical density of the western blot results and to calculate the number of caspase-1-positive cells or double staining cells and JC-1-stained cells. Statistical analyses were performed using the SPSS 19.0 (SPSS Inc., USA) software. Data were presented as the means ± SEM with the homogeneity of variance. Statistical analyses were performed with Student’s *t* test between two groups or one-way ANOVA for multiple groups, followed by LSD for post hoc comparisons. Two-way ANOVA was used to compare the results among multiple groups according to the immunofluorescence in the brain slices. *p* < 0.05 was considered statistically significant.

## Results

### The cellular location where NLRP3 inflammasomes were activated changed dynamically in the process of ischemia/reperfusion (I/R) injury

It was observed in the ischemic core area (Fig. 1f) that cleaved caspase-1 was mainly expressed in microglia 6 h after the I/R injury (88.36 ± 1.102%) (Fig. 1a) and was rarely expressed in other cell types (Fig. 1b–d). Then, cleaved caspase-1 was mostly expressed in neurons (63.39 ± 2.219%) (Fig. 1c) and endothelial cells (39.97 ± 2.289%) (Fig. 1d) at 24 h, while limited expressed in microglia (12.21 ± 1.068%) (Fig. 1a) and astrocytes (4.67 ± 0.985%) (Fig. 1b) was observed. Therefore, the expression of cleaved caspase-1 gradually decreased in microglia between 6 h and 24 h but simultaneously increased in neurons and endothelial cells, particularly in neurons.

**Fig. 2:**
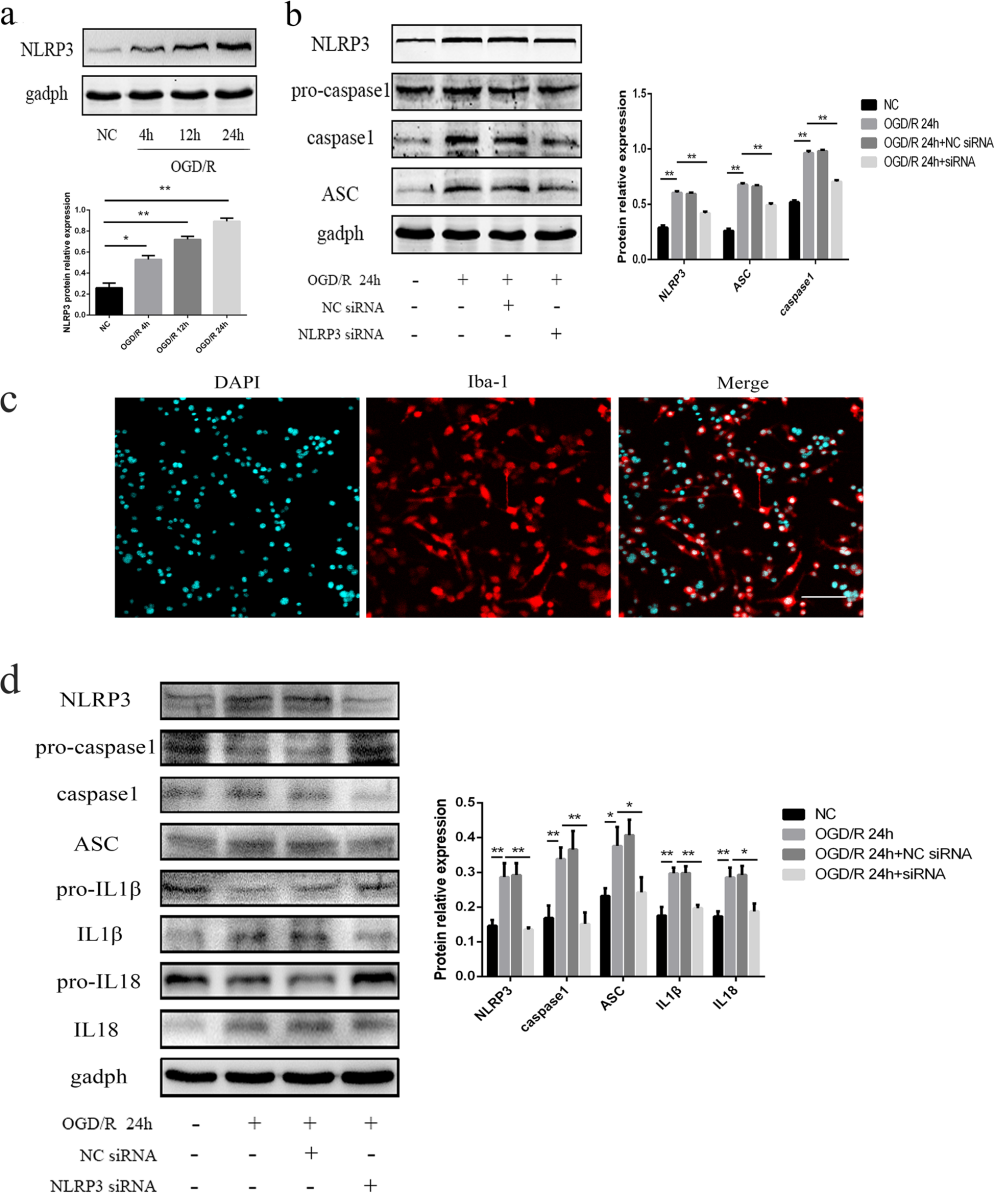
The expression of NLRP3 inflammasomes in BV2 cells or primary microglia, as measured by western blot. **a** The expression of NLRP3 in BV2 cells at different time points after reoxygenation. **b** The expression of NLRP3, ASC, pro-caspase-1, and cleaved caspase-1 in BV2 cells among the different groups. **c** The purity of primary microglia. **d** The expression of NLRP3, ASC, pro-caspase-1, cleaved caspase-1, pro-IL1β, cleaved IL1β, pro-IL18, and cleaved IL18 in primary microglial cells among the different groups. OGD/R treatment was conducted at 24 h after siRNA transfection. Bar = 100 μm. *p < 0.05, **p < 0.01. OGD/R: oxygen-glucose deprivation/reoxygenation. The OGD continued for 4 h, followed by reoxygenation

As cleaved caspase-1 was expressed in microglia first, we used primary microglia and BV2 cells to explore the pathway of caspase-1 activation. The purity of primary microglia was verified as 95% or higher (Fig. 2c). We found that the protein level of NLRP3 in BV2 cells increased over time after OGD/R, especially at 24 h, compared with the NC (all *p* < 0.01) (Fig. 2a). When NLRP3 expression was silenced by NLRP3-siRNA transfection in the primary microglia and BV2 cells, we observed that the expression levels of cleaved caspase-1, ASC, cleaved IL-1β, and cleaved IL-18 were significantly downregulated after OGD/R (*p* < 0.05) (Fig. 2b and d), indicating that the inflammasome pathway that was activated in microglia was mediated by NLRP3, and the activation of NLRP3 inflammasomes in BV2 cells after OGD/R could reflect the changes in primary microglia.

### NLRP3 inflammasomes expressed in PC12 and bEnd3 cells after OGD/R were induced by co-cultured BV2 cells

**Fig. 3:**
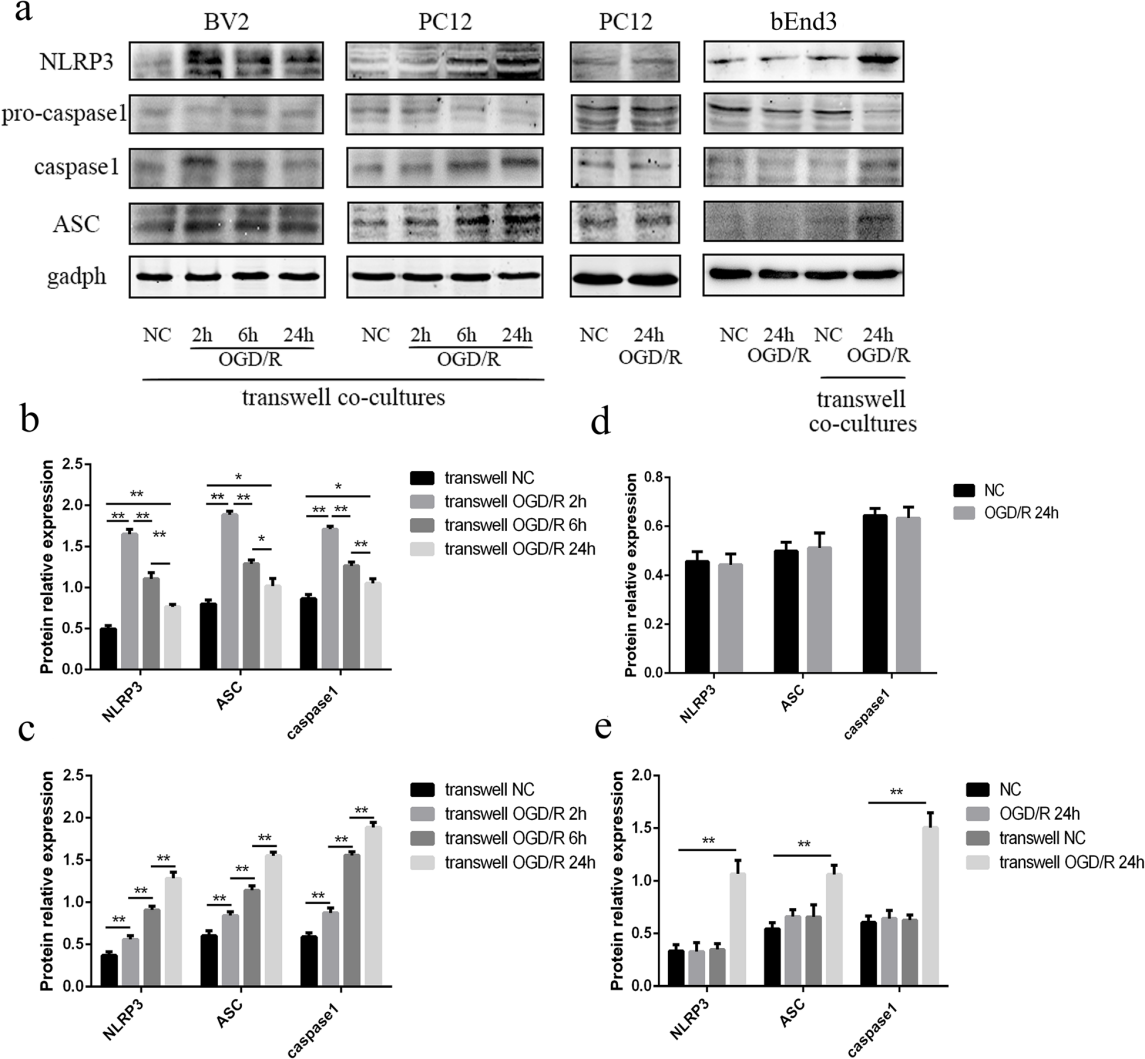
NLRP3 inflammasome pathway expressed in PC12 and bEnd3 cells in the transwell co-culture system after OGD/R was induced by BV2 cells. **a** The expression levels of NLRP3, ASC, pro-caspase-1, and cleaved caspase-1 in BV2, PC12, and bEnd3 cells in different groups, as measured by western blot. **b** The changes of NLRP3, ASC, and cleaved caspase-1 in BV2 cells in the transwell co-culture with PC12 cells at different time points after reoxygenation, as measured by western blot. **c** The changes of NLRP3, ASC, and cleaved caspase-1 in PC12 cells in the transwell co-culture with BV2 cells at different time points after reoxygenation, as measured by western blot. **d** The changes of NLRP3, ASC, and cleaved caspase-1 in PC12 cells with isolated cultured between the two groups, as measured by western blot. **e** The changes of NLRP3, ASC, and cleaved caspase-1 in bEnd3 cells among the different groups, as measured by western blot. *p < 0.05, **p < 0.01. OGD/R: oxygen-glucose deprivation/reoxygenation. The OGD continued for 4 h, followed by reoxygenation

PC12 and bEnd3 cells were used as alternative neurons and vascular endothelial cells, respectively. A transwell co-culture system, with BV2 cells in the upper chamber and PC12 or bEnd3 cells in the lower chamber, was used to explore the expression of NLRP3 inflammasomes in PC12 and bEnd3 cells. The results showed that the expression of NLRP3 was roughly upregulated in BV2 cells 2 h after OGD/R compared with the NC group in the transwell co-culture system of BV2 and PC12 cells (*p* < 0.01); this expression gradually decreased over time (all *p* < 0.01). In addition, the same trend was observed in ASC and cleaved caspase-1 in the BV2 cells co-cultured with PC12 cells (all *p* < 0.05) (Fig. 3a and b). In contrast, we found that the expression levels of NLRP3, ASC, and cleaved caspase-1 all increased over time in PC12 cells after OGD/R in the transwell co-culture system (all *p* < 0.01) (Fig. 3a, c). However, the activation of NLRP3 inflammasomes was not observed in PC12 cells cultured alone after OGD/R (Fig. 3a, d). Similarly, the expression levels of NLRP3, ASC, and cleaved caspase-1 were significantly upregulated in bEnd3 cells co-cultured with BV2 cells 24 h after OGD/R, compared with the transwell NC group, OGD/R group, and NC group (all *p* < 0.01). The above results indicated that NLRP3 inflammasome activation in PC12 and bEnd3 cells in the transwell co-culture system after OGD/R was induced by some stimulating factor released from BV2 cells.

**Fig. 4:**
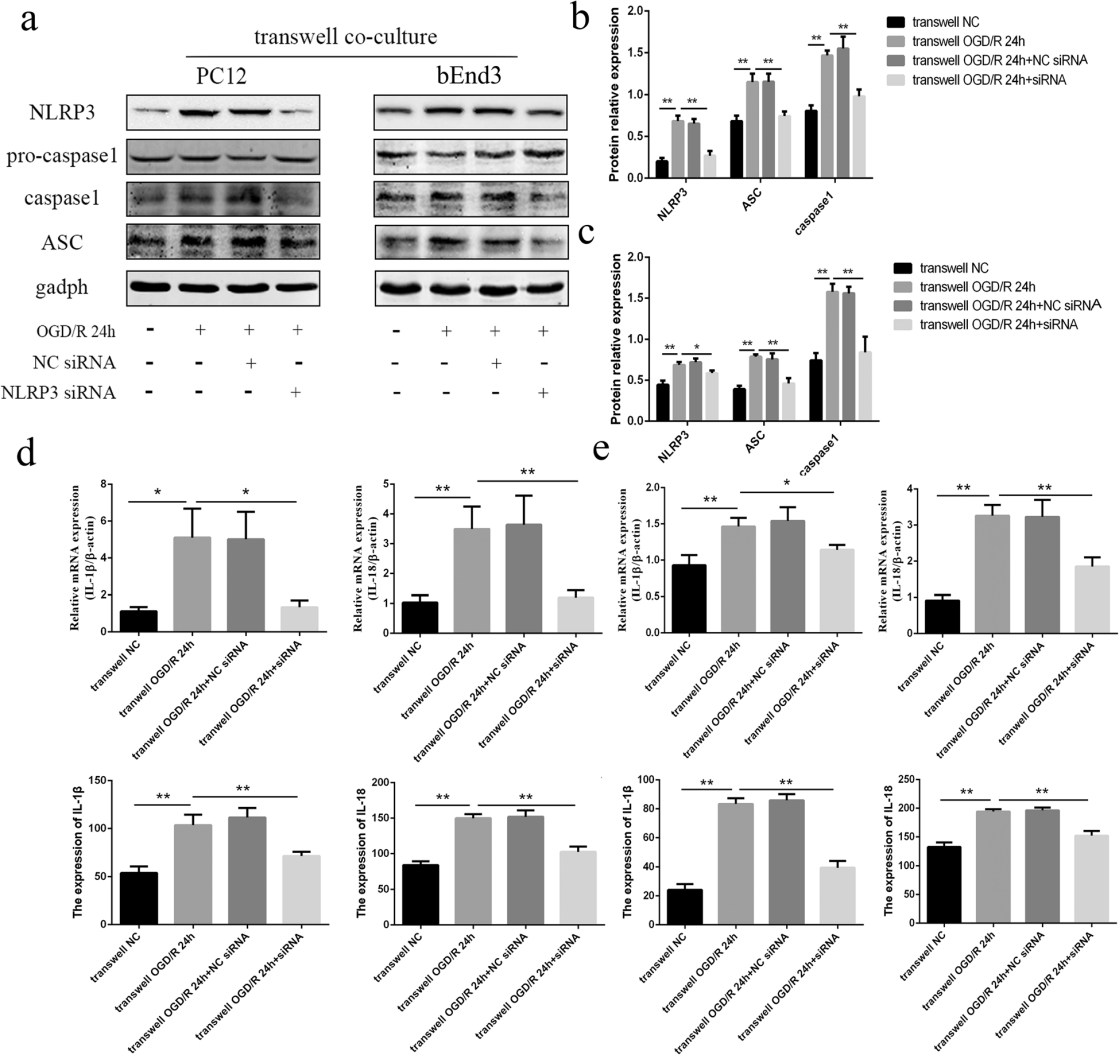
The stimuli released from co-cultured BV2 cells originated from NLRP3 inflammasome signaling pathway in BV2 cells. **a** The expression levels of NLRP3, ASC, pro-caspase-1, and cleaved caspase-1 in PC12 and bEnd3 cells in the transwell co-cultures in different groups, as measured by western blot. OGD/R treatment of transwell co-culture system was conducted at 24 h after siRNA transfection in BV2 cells. **b** The changes of NLRP3, ASC, and cleaved caspase-1 in PC12 cells among the different groups, as measured by western blot. **c** The changes of NLRP3, ASC, and cleaved caspase-1 in bEnd3 cells among the different groups, as measured by western blot. **d** The expression levels of IL-1β and IL-18 in PC12 cells among the different groups, as measured by RT-PCR and ELISA. **e** The expression levels of IL-1β and IL-18 in bEnd3 cells among the different groups, as measured by RT-PCR and ELISA. *p < 0.05, **p < 0.01. OGD/R: oxygen-glucose deprivation/reoxygenation. The OGD continued for 4 h, followed by reoxygenation

Then, we transfected NLRP3-siRNA to block the production of NLRP3 in BV2 cells, followed by co-cultures with PC12 or bEnd3 cells and OGD/R treatment. It was observed that the expression levels of NLRP3, ASC, and cleaved caspase-1 in PC12 cells were significantly downregulated in the transwell OGD/R + NLRP3-siRNA group compared to the transwell OGD/R group and transwell OGD/R + NC-siRNA group (Fig. 4a and b) (all *p* < 0.01). Furthermore, the results from the qRT-PCR and ELISAs also revealed that the upregulation of IL-1β and IL-18 in the PC12 cells, and supernatants induced by OGD/R were both rescued by NLRP3-silencing in the BV2 cells (Fig. 4d) (all *p* < 0.05). The same trend was also observed in bEnd3 cells (Fig. 4a, c, e). These results indicated that the stimuli released from BV2 cells were originated from the activated NLRP3 inflammasome signaling pathway in BV2 cells.

**Fig. 5:**
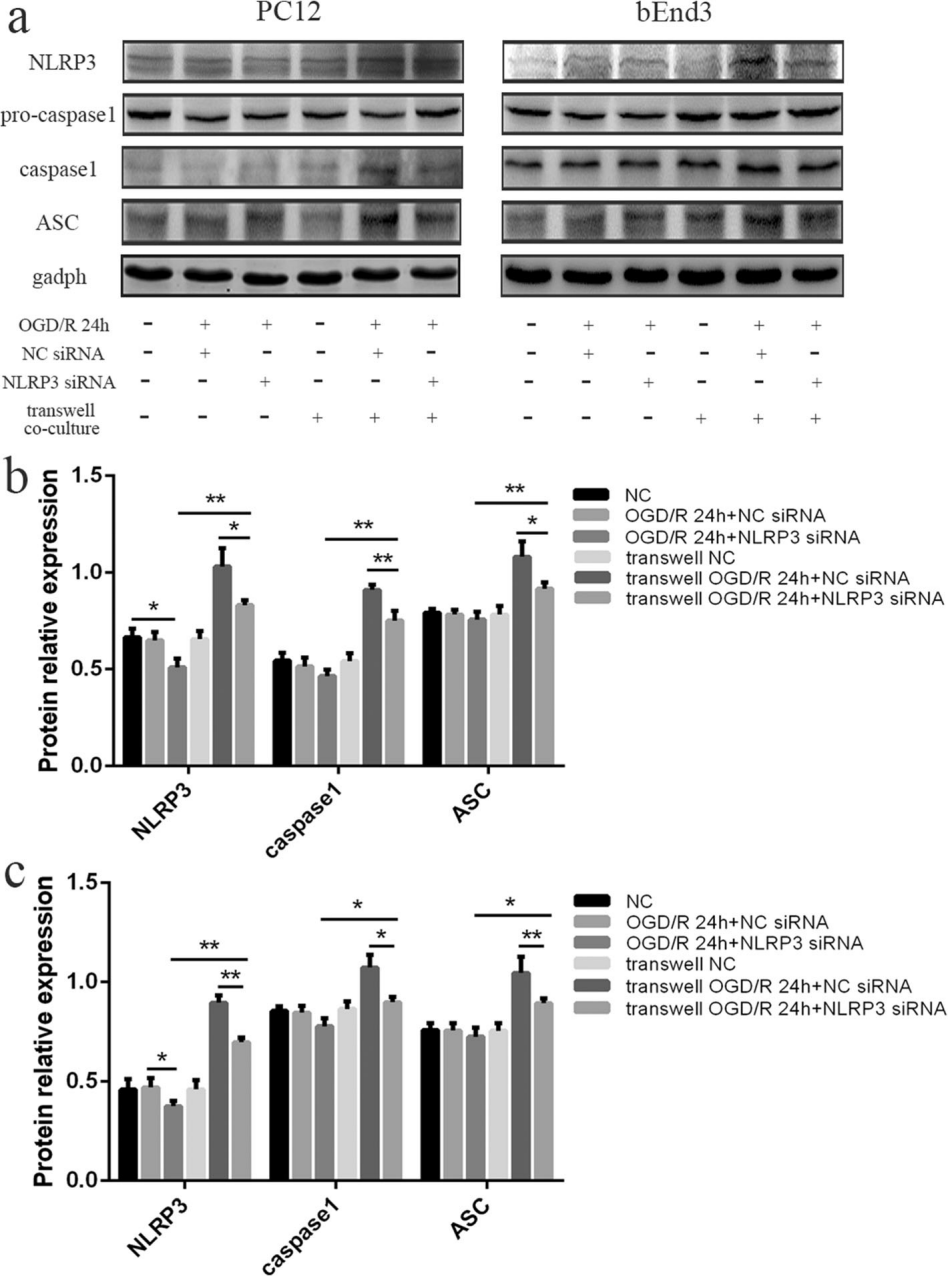
The main source of NLRP3 inflammasomes expressed in PC12 or bEnd3 cells in transwell co-culture system was from their own productions. **a** The expression levels of NLRP3, ASC, pro-caspase-1, and cleaved caspase-1 in PC12 and bEnd3 cells in different groups, as measured by western blot. OGD/R treatment in isolated cultures or transwell co-cultures was conducted at 24 h after siRNA transfection in PC12 or bEnd3 cells. **b** The changes of NLRP3, ASC, and cleaved caspase-1 in PC12 cells among the different groups, as measured by western blot. **c** The changes of NLRP3, ASC, and cleaved caspase-1 in bEnd3 cells among the different groups, as measured by western blot. *p < 0.05, **p < 0.01. OGD/R: oxygen-glucose deprivation/reoxygenation. The OGD continued for 4 h, followed by reoxygenation

To clarify the source of NLRP3 expressed in PC12 or bEnd3 cells, we transfected NLRP3-siRNA to block the production of NLRP3 in PC12 or bEnd3 cells, followed by co-cultures with BV2 cells and OGD/R treatment. The results show that the expression of NLRP3, ASC, and cleaved caspase-1 in PC12 (Fig. 5a and b) or bEnd3 cells (Fig. 5a, c) in the transwell OGD/R + NLRP3-siRNA group decreased significantly compared to the transwell OGD/R + NC-siRNA group, but were still higher than that in OGD/R + NLRP3-siRNA group (all *p* < 0.05). Such results reinforced the finding that the activation of NLRP3 inflammasomes in PC12 or bEnd3 cells was induced by BV2 cells, and revealed that the main source of NLRP3 inflammasomes expressed in PC12 or bEnd3 cells was from their own productions.

### NLRP3 inflammasomes in BV2 cells after OGD/R were associated with BV2 cell-mediated PC12 cell damage

**Fig. 6:**
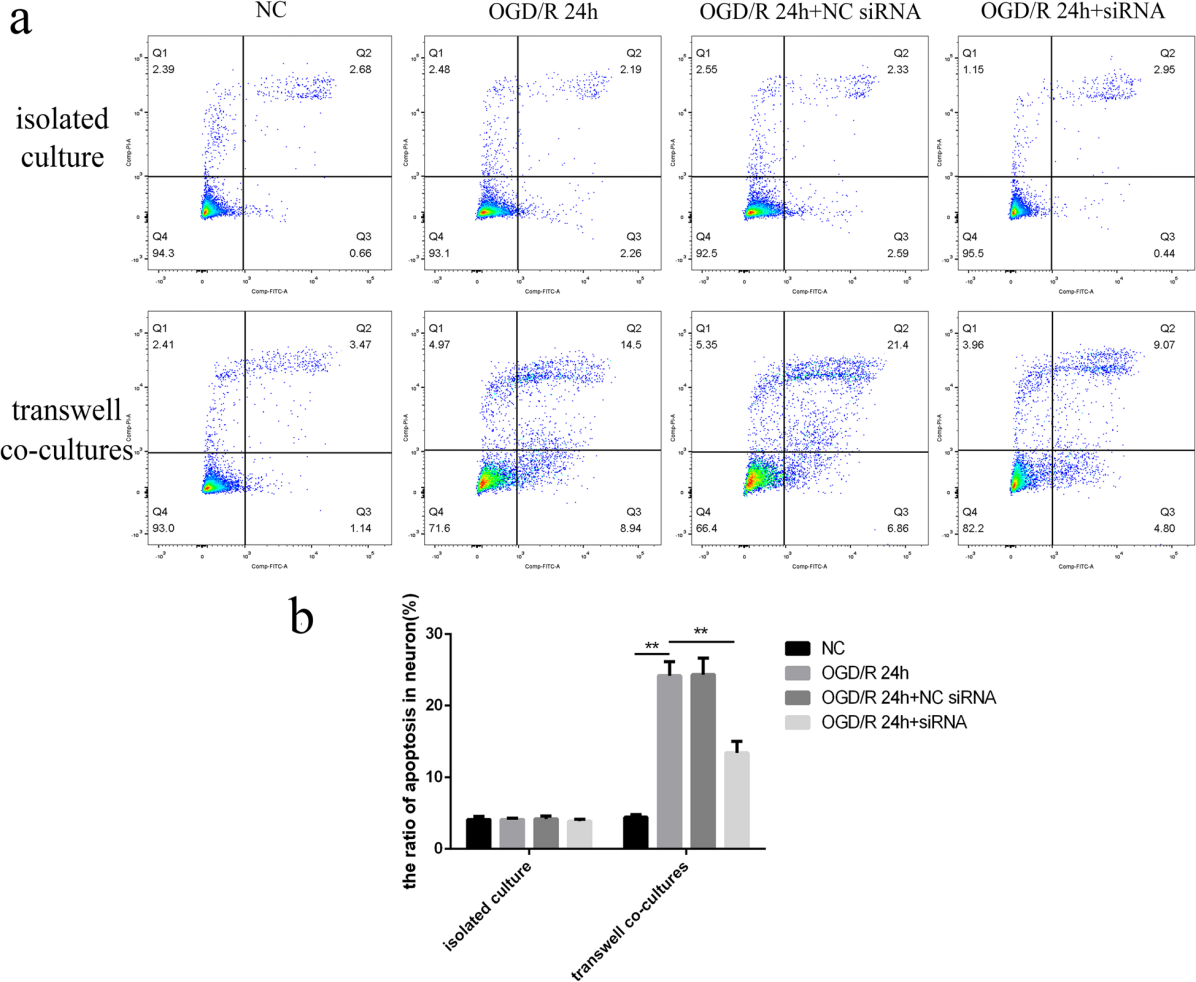
NLRP3 knockdown in BV2 cells could inhibit the apoptosis of PC12 cells in the transwell co-cultures after OGD/R. **a, b** The rate of apoptotic PC12 cells among the different groups, as measured by flow cytometry. OGD/R treatment in isolated cultures or transwell co-cultures was conducted at 24 h after siRNA transfection. *p < 0.05, **p < 0.01. OGD/R: oxygen-glucose deprivation/reoxygenation. The OGD continued for 4 h, followed by reoxygenation

The results from the flow cytometry (Fig. 6a) showed that BV2 cells could significantly increase the apoptotic rate of PC12 cells after OGD/R (24.19 ± 1.948%), compared with the NC group (4.44 ± 0.348%). The apoptosis of PC12 cells in transwell co-cultures after OGD/R could be attenuated via NLRP3 knockdown in BV2 cells (13.43 ± 1.594%), indicating that the damage to PC12 cells was induced by the NLRP3 inflammasomes in BV2 cells after OGD/R (all *p* < 0.01) (Fig. 6b). Such results were not shown in isolated cultured PC12 cells after OGD/R.

### Mitochondrial dysfunction could activate NLRP3 inflammasomes in primary microglia and BV2 cells after OGD/R

**Fig. 7:**
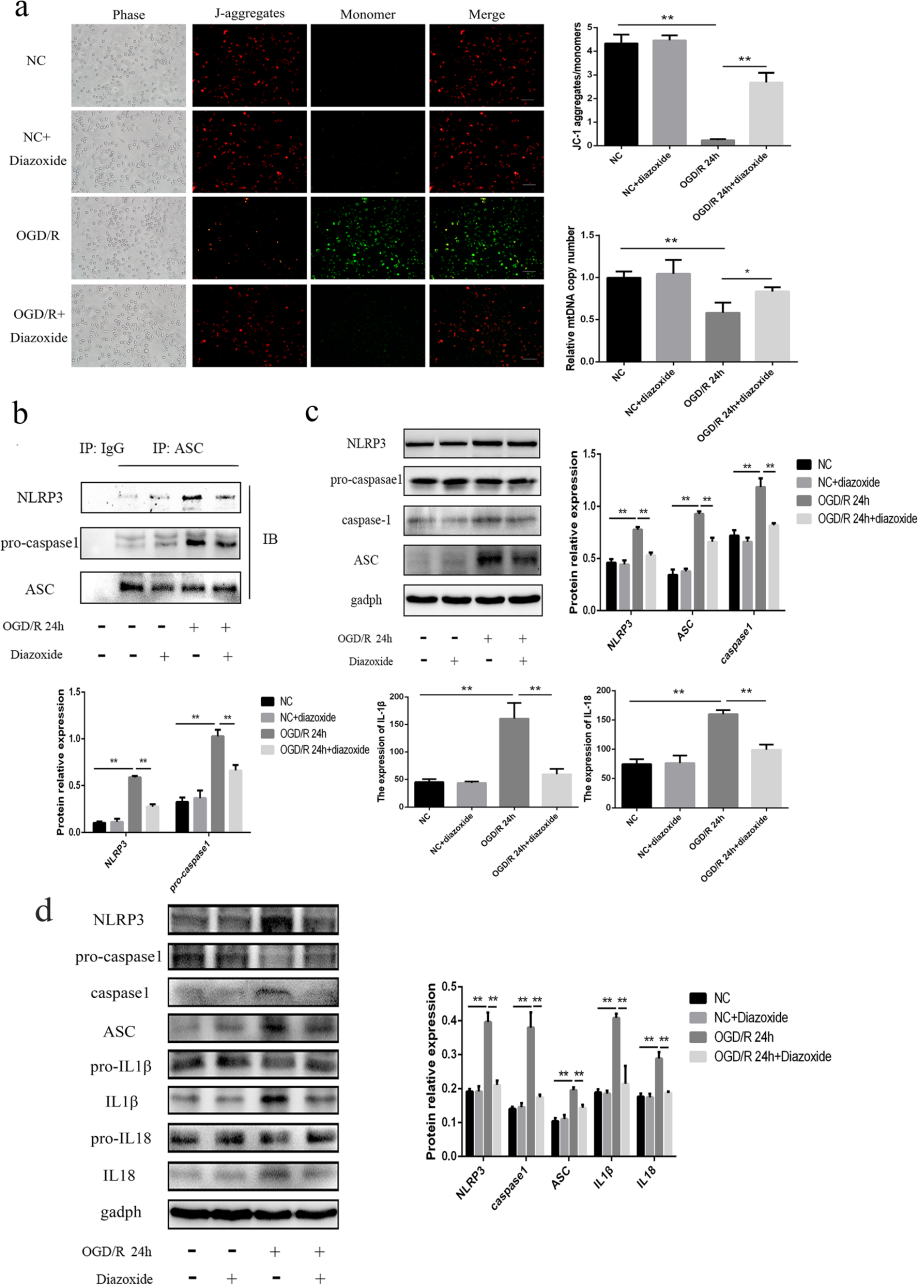
Mitochondrial dysfunction could activate NLRP3 inflammasomes in BV2 cells after OGD/R. **a** Measurements of the ∆ψm and mtDNA copy numbers in BV2 cells among the different groups. The BV2 cells in OGD/R or OGD/R + diazoxide groups were measured at 24 h after reoxygenation. **b** The expression levels of NLRP3 and pro-caspase-1 physically associated with ASC in BV2 cells among the different groups, as measured by IP. Anti-ASC antibody was used to immunoprecipitate NLRP3 inflammasome. IB assay for ASC was used as a loading control. **c** The expression levels of NLRP3, ASC, cleaved caspase-1, IL-1β, and IL-18 in BV2 cells among the different groups, as measured by western blot and ELISA. **d** The expression of NLRP3, ASC, pro-caspase-1, cleaved caspase-1, pro-IL1β, cleaved IL1β, pro-IL18, and cleaved IL18 in primary microglial cells among the different groups. The 100 μm diazoxide was applied to the cells when got reoxygenation. Bar = 100 μm. *p < 0.05, **p < 0.01. OGD/R: oxygen-glucose deprivation/reoxygenation. The OGD continued for 4 h, followed by reoxygenation

We found that the ratio of JC-1 aggregates to monomers and the mtDNA copy numbers significantly decreased in BV2 cells 24 h after OGD/R compared to the NC group (all *p* < 0.01) (Fig. 7a), which indicated damage to the mitochondria, including mitochondrial depolarization and mtDNA damage. In addition, this damage to the mitochondria was able to be rescued by the mitochondrial protector diazoxide (all *p* < 0.01) (Fig. 7a).

Next, we aimed to clarify the relationship between mitochondrial dysfunction and NLRP3 inflammasomes. For detecting the formation of NLRP3 inflammasome, which consists of NLRP3, ASC, and pro-caspase1, IP assay was performed, which was the direct evidence for protein interaction. The IP results revealed that the same amount of ASC in BV2 cells in OGD/R 24-h group could bind more NLRP3 and pro-caspase1 compared to the NC, indicating that the formation of the NLRP3 inflammasomes was obviously increased in BV2 cells after OGD/R, which could be inhibited by diazoxide (all *p* < 0.01) (Fig. 7b). In addition, diazoxide could also suppress the upregulation of NLRP3, ASC, and cleaved caspase-1induced by OGD/R in primary microglia and BV2 cells (Fig. 7c and d) (all *p* < 0.01). We also found that the upregulation of IL-1β and IL-18 24 h after OGD/R was suppressed by diazoxide in primary microglia and BV2 cells (all *p* < 0.01) (Fig. 7c and d). These results suggested that mitochondrial dysfunction was essential for the NLRP3 inflammasome formation and activation in primary microglia and BV2 cells after OGD/R. Also, the pathway in BV2 cells was same with primary microglia.

### The mitochondrial protector could rescue the NLRP3 inflammasome pathway expressed in PC12 and bEnd3 cells in transwell co-cultures after OGD/R

**Fig. 8:**
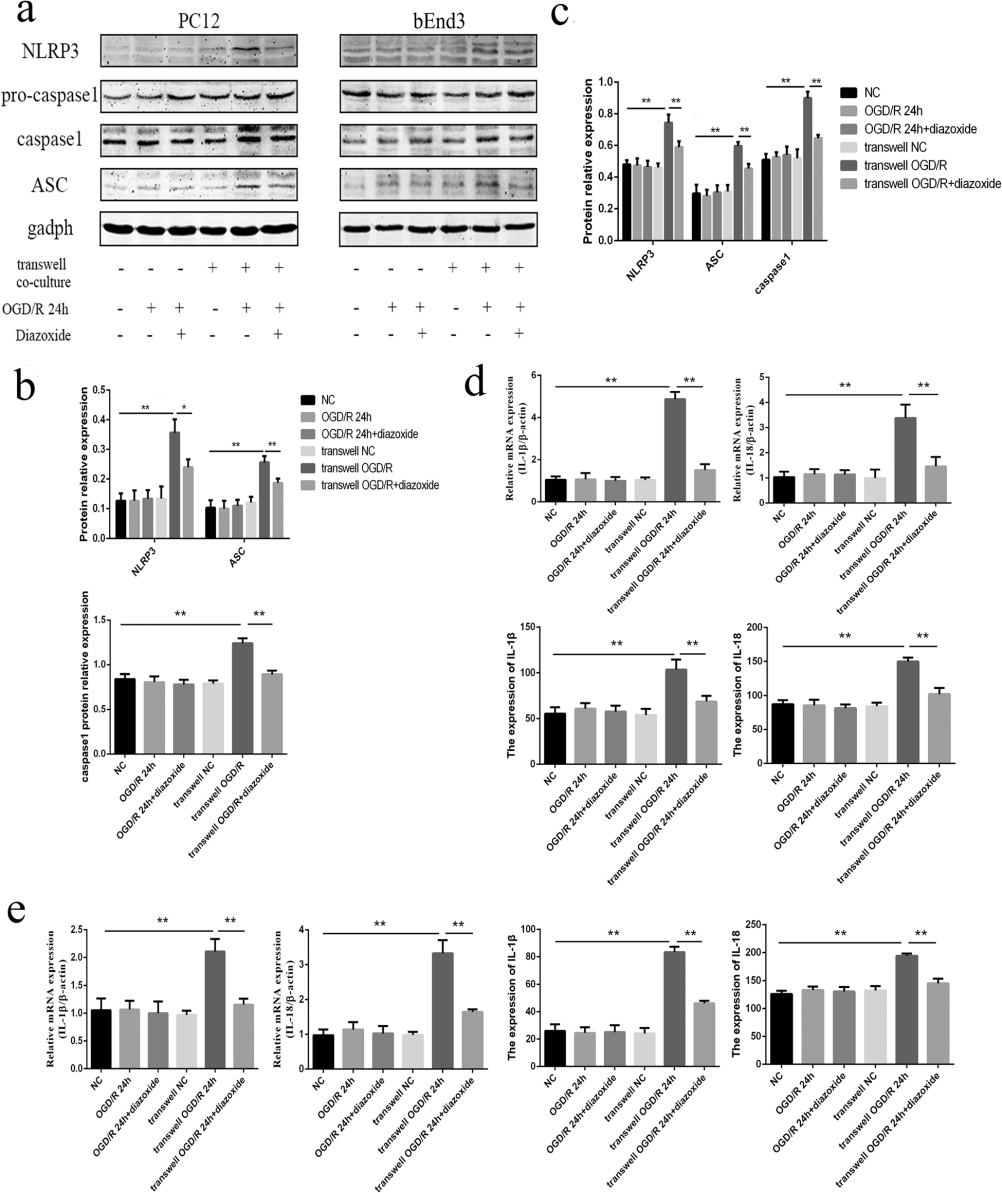
Mitochondrial protector could rescue the NLRP3 inflammasome pathway expressed in PC12 and bEnd3 cells in the transwell co-cultures after OGD/R. **a** The expression levels of NLRP3, ASC, pro-caspase-1, and cleaved caspase-1 in PC12 and bEnd3 cells among the different groups, as measured by western blot. **b** The changes of NLRP3, ASC, and cleaved caspase-1 in PC12 cells among the different groups, as measured by western blot. **c** The changes of NLRP3, ASC, and cleaved caspase-1 in bEnd3 cells among the different groups, as measured by western blot. **d** The expression levels of IL-1β and IL-18 in PC12 cells among the different groups, as measured by RT-PCR and ELISA. **e** The expression levels of IL-1β and IL-18 in bEnd3 cells among the different groups, as measured by RT-PCR and ELISA. The 100 μm diazoxide was applied to the cells when got reoxygenation. *p < 0.05, **p < 0.01. OGD/R: oxygen-glucose deprivation/reoxygenation. The OGD continued for 4 h, followed by reoxygenation

The WB results revealed that the protein levels of NLRP3, ASC, and cleaved caspase-1 significantly decreased in PC12 (Fig. 8a and b) and bEnd3 cells (Fig. 8a, c) in the transwell OGD/R 24 h + diazoxide group, compared with the transwell OGD/R 24-h group (all *p* < 0.05). In addition, the results from the qRT-PCR and ELISA analyses also showed that the expression levels of IL-1β and IL-18 in PC12 (Fig. 8d) and bEnd3 cells (Fig. 8e) were all significantly downregulated in the transwell OGD/R 24 h + diazoxide group compared with the transwell OGD/R 24-h group (all *p* < 0.01). The other control groups showed no obvious changes compared with the NC groups. Such results indicated that the mitochondrial protector could effectively alleviate the NLRP3 inflammasome response in BV2, PC12, and bEnd3 cells after OGD/R.

### The mitochondrial protector could inhibit the activation of NLRP3 inflammasome in cerebral I/R injury

**Fig. 9:**
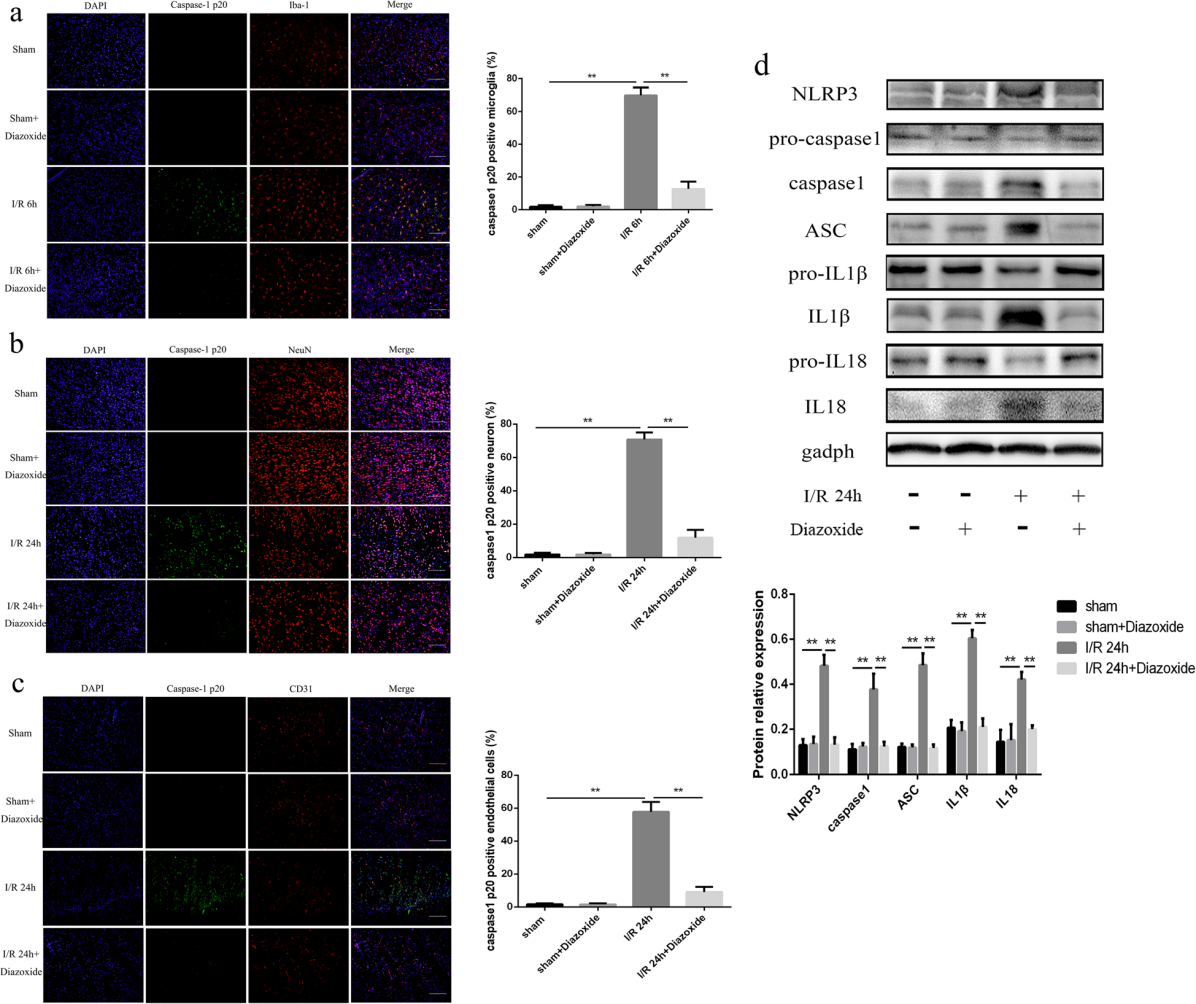
The mitochondrial protector could inhibit the activation of NLRP3 inflammasome in cerebral I/R injury. **a–c** The expression of caspase-1 p20 in microglia, neurons, and endothelial cells in sham, sham+diazoxide, I/R, I/R + diazoxide groups. **d** The expression of NLRP3, ASC, pro-caspase-1, cleaved caspase-1, pro-IL1β, cleaved IL1β, pro-IL18, and cleaved IL18 in different groups. The 10 mg/kg diazoxide was treated intraperitoneally when the rats got reperfusion. Bar = 100 μm. *p < 0.05, **p < 0.01

The results of IF showed that the expression of cleaved caspase-1 was significantly decreased in microglia (12.72 ± 1.806% VS 69.9 ± 1.957%, Fig. 9a), neurons (11.87 ± 1.933% VS 70.76 ± 1.737%, Fig. 9b), and endothelial cells (9.12 ± 1.278% VS 57.76 ± 2.455%, Fig. 9c) in I/R + diazoxide groups at 6 h or 24 h after the cerebral I/R injury, compared with the I/R group (all *p* < 0.01).

The WB results revealed that the protein levels of NLRP3, ASC, cleaved caspase-1, cleaved IL-1β, and cleaved IL-18 significantly increased in I/R 24-h group, compared with the sham group (all *p* < 0.01) (Fig. 9d), and diazoxide could significantly inhibit the expression of NLRP3, ASC, cleaved caspase-1, cleaved IL-1β, and cleaved IL-18 (all *p* < 0.01) (Fig. 9d). These results showed that the mitochondrial protector could inhibit the activation of NLRP3 inflammasome in cerebral I/R injury, indicating that mitochondrial dysfunction played a great role in activating NLRP3 inflammasome in cerebral I/R injury.

## Discussion

Recently, NLRP3 inflammasomes have been found in some organs after I/R injury, such as the brain, heart, kidneys, and testes [19]. As has been reported, damage-associated molecular pattern (DAMP) is the critical initial stimulus to activate NLRP3 [20]. The oligomerization of NLRP3 recruits, ASC and ASC, could activate pro-caspase-1 to cleave into active fragments [21], and then cleaved caspase-1 induces the formation of mature pro-inflammatory cytokines, namely, IL-1β and IL-18 [22, 23]. These cytokines then initiate or amplify diverse downstream signaling pathways to drive pro-inflammatory processes [24], leading to cellular damage, such as autophagy and pyroptosis [22], which could release DAMPs to induce more inflammation [25].

In the mouse brain, it has been observed that NLRP3, ASC, and caspase-1 are expressed in microglia after LPS stimulation, which was not detected in astrocytes, indicating that microglia might be the main site involved in the formation of functional NLRP3 inflammasomes [4]. Moreover, Fann et al. and Wang et al. discovered that the levels of NLRP3 inflammasome proteins and of IL-1β and IL-18 were upregulated in primary cortical neurons under OGD/R conditions [5, 26]. In contrast, in a mouse model of middle cerebral artery occlusion (MCAO), Yang et al. demonstrated that NLRP3 was expressed in microglia and vascular endothelial cells but not in neurons [6]. Thus, the specific expression and distribution of NLRP3 inflammasomes in cerebral I/R injury have not led to a conclusion. In addition, the different models of ischemia, the durations of the ischemic insults, and the different interventions have been offered as possible explanations.

In this study, we chose rats as the animal model because the genes of rats are relatively close to those of humans. In addition, it was the first time the cellular localization of the NLRP3 inflammasome pathway after I/R injury was observed dynamically. The cells used in vitro were primary microglia, BV2 microglia, PC12 neurons, and bEnd3 cerebral microvascular endothelial cells. We found that the cleaved caspase-1 in tMCAO rats was mainly expressed in ischemic core area within 24 h, which was the observed and counted area. Our results showed that activated inflammasomes were first formed in microglia after cerebral I/R injury, but not in astrocytes, and then they were mostly expressed in neurons and vascular endothelial cells at 24 h, particularly in neurons, mainly through the NLRP3 molecules. The results of this in vitro study revealed that the NLRP3 inflammasome pathway expressed in PC12 and bEnd3 cells in transwell co-culture systems after OGD/R was induced by BV2 cells, as the levels of NLRP3 inflammasomes were not changed in PC12 and bEnd3 cells only under OGD/R condition. Then, we used NLRP3-siRNA to knockdown the target gene in BV2 cells, which were cultured in a transwell co-culture system, thereby inducing a rough decrease in the level of NLRP3 inflammasomes and downstream inflammatory factors, such as caspase-1, IL-1β, and IL-18 in PC12 and bEnd3 cells in a transwell co-culture system after OGD/R. When we inhibited the expression of NLRP3 in PC12 or bEnd3 cells which were cultured in transwell co-culture system, the increase of NLRP3 inflammasomes in PC12 and bEnd3 cells in transwell co-culture system after OGD/R could be inhibited partly. These findings provided positive proof supporting that the NLRP3 inflammasomes that were expressed in PC12 and bEnd3 cells in transwell co-culture systems after OGD/R were activated by some stimulating factor originated from NLRP3 inflammasome signaling pathway in BV2 cells, and the main source of NLRP3 inflammasomes expressed in PC12 or bEnd3 cells was from their own productions. Furthermore, the apoptosis of PC12 cells was clearly activated when these cells were cultured with BV2 cells after OGD/R, which could be inhibited by NLRP3 knockdown in BV2 cells. A previous study revealed that NLRP3 inflammasomes induced caspase-1-dependent pyroptosis, which is an important event that may be an essential pathway involved in mitochondria-associated apoptosis in ketamine-induced hippocampal neurotoxicity [27]. These previous results were in accordance with our results. Taken together, it is reasonable to draw the conclusion that microglia are the main source of activated NLRP3 inflammasomes during the early stage after cerebral I/R injury, which could drive pro-inflammatory processes, leading to the cells death and disruption, which could release some stimuli (e.g., DAMPs and IL-1β) to trigger the activation of inflammasomes in surrounding cells [28, 29]. Thus, the NLRP3 inflammasomes are activated in neurons and microvascular endothelial cells over time and mainly gather in neurons during the late stage, which may induce neuronal death and blood-brain barrier (BBB) integrity dysfunction. Interestingly, we found that the expression of NLRP3 inflammasome in BV2 cells in co-culture system was gradually decreased over time, which was inconsistent with the results of isolated cultured BV2 cells. We thought the different cellular models were the main reason. In co-culture model, the released DAMP could be spread to the bottom chambers, which may result in the attenuation of activation of NLRP3 inflammasome in BV2 cells, compared to isolated culture model. Besides, the PC12 cells in co-culture model, expressing NLRP3 inflammasome later, may release some negative feedback regulator, which may inhibit the expression of NLRP3 inflammasome in BV2 cells. Of course, the specific reason still needs further studies.

As we know, microglia and astrocytes are the main cells inducing immunoreaction to cerebral I/R injury. In our results, the quantity of microglia and astrocytes in ischemic core area showed a severe decrease, especially at 24 h after cerebral I/R injury, but the microglia were activated significantly. Some studies have revealed that a specific loss of GFAP immunolabeling in protoplasmic astrocytes occurred in the area with total depletion of regional CBF (rCBF) levels, associated with advanced disintegration of cytoplasmic elements and loss of ATP in the ischemic core, whereas “classical” astrogliosis was observed in areas with remaining rCBF [30, 31], which was in accordance with our results. Also, the findings in the ischemic core, the round Iba1-positive cells appeared from 24 h and reached a peak at 4 to 7 days [32], could support our results, and degeneration of microglia in the ischemic core after prolonged MCAO was also reported by other studies [33, 34].

A series of studies have demonstrated that NLRP3 inflammasomes are activated by three mechanisms: the potassium efflux [35], the release of mitochondrial reactive oxygen species (mtROS) [36], and lysosomal damage [37]. However, it has been suggested that these three models could be integrated and associated with the production of oxidized mitochondrial DNA (mtDNA) [38, 39]. Zhou et al. reported that ROS that were generated following mitochondrial dysfunction, which manifested as a decrease in the mitochondrial membrane potential (∆ψm), induced by the opening of the mitochondrial permeability transition pore (mPTP), could promote NLRP3 inflammasome activation [40]. In addition to the mtROS, mtDNA released into the cytoplasm from the damaged mitochondria has also been proposed to act as a mitochondrial danger signal, promoting the activation of NLRP3 inflammasomes [38, 41]. Similarly, we found that the function of mitochondria in BV2 cells after OGD/R was damaged and included mitochondrial depolarization and mtRNA damage. In addition, diazoxide was shown to be able to protect the function of mitochondria by preventing mitochondrial depolarization and mtRNA damage, which aligned with previous studies that discovered that diazoxide could prevent the opening of the mPTP and mitochondrial depolarization in cardiac hypertrophy and in an oxidatively stressed ischemic environment [42, 43]. Thus, we used diazoxide to determine the relationship between mitochondrial dysfunction and NLRP3 inflammasomes in primary microglia and BV2 cells. The results indicated that mitochondrial dysfunction played a substantial role in the activation of the NLRP3 inflammasome pathway in primary microglia and BV2 cells after OGD/R, and that diazoxide could effectively alleviate the NLRP3 inflammasome response in primary microglia and BV2 cells, transwell co-cultured PC12, and bEnd3 cells after OGD/R. Then we detected the activation of NLRP3 inflammsome pathway in rats during cerebral I/R injury, and found that the diazoxide could inhibit the activation of NLRP3 inflammasome, which indicated that mitochondrial dysfunction played a great role in activating NLRP3 inflammasome in cerebral I/R injury.

## Conclusion

Our study was the first to find the dynamic change in the cellular localization of the NLRP3 inflammasome pathway after cerebral I/R injury, showing that microglia are the main source of activated NLRP3 inflammasomes during the early stage after cerebral I/R injury, and then the NLRP3 inflammasomes were activated in neurons and microvascular endothelial cells over time and mainly gathered in neurons during the late stage. Furthermore, mitochondrial dysfunction was essential for the activation of NLRP3 inflammasomes in microglia, and a mitochondrial protector could effectively alleviate the NLRP3 inflammasome response in microglia, neurons, and microvascular endothelial cells after OGD/R and cerebral I/R injury.

## Additional files

Additional file 1: **Figure S1.** The confirmation of the CD31 antibody specificity. The bEnd3 cells were used as positive cells. The double staining cells of vWF and CD31 were up to 98%. Bar = 100 μm. (TIF 947 kb)

Additional file 2: **Figure S2.** The morphology of differential PC12 cells. (JPG 2204 kb)

Additional file 3: **Figure S3.** Ethics approval of the animal usage. (JPG 1160 kb)

## Abbreviations

∆ψm: Mitochondrial membrane potential
ASC: Apoptosis-associated speck-like protein containing a caspase activation recruitment domain
BBB: Blood-brain barrier
I/R: Ischemia/reperfusion
IF: Immunoprecipitation
mPTP: Mitochondrial permeability transition pore
mtDNA: Mitochondrial DNA
mtROS: Mitochondrial reactive oxygen species
NLRP3: Nod-like receptor protein 3
OGD/R: Oxygen-glucose deprivation/reoxygenation
pro-caspase1: Precursor caspase-1
qPCR: Real-time quantitative PCR
tMCAO: Transient middle cerebral artery occlusion
