# Supplementary figures and images for: Mitochondrial dysfunction induces NLRP3 inflammasome activation during cerebral ischemia/reperfusion injury
Source: J Neuroinflammation. 2018 Aug 28;15:242. doi: 10.1186/s12974-018-1282-6 (PMC6114292; doi:10.1186/s12974-018-1282-6)

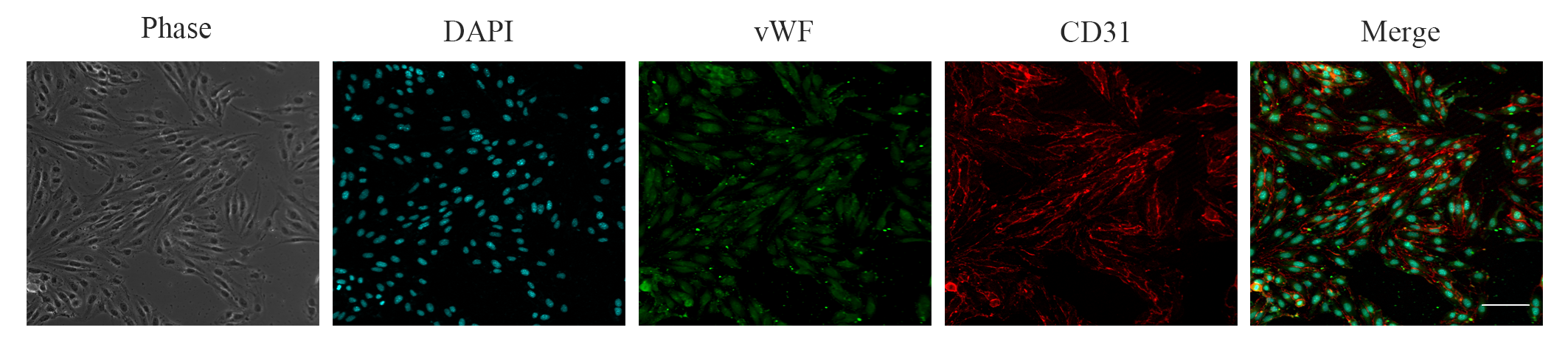

Supplement: Supplementary file 1 — Figure S1. The confirmation of the CD31 antibody specificity. The bEnd3 cells were used as positive cells. The double staining cells of vWF and CD31 were up to 98%. Bar = 100 μm. (TIF 947 kb) [file 12974_2018_1282_MOESM1_ESM.tif]

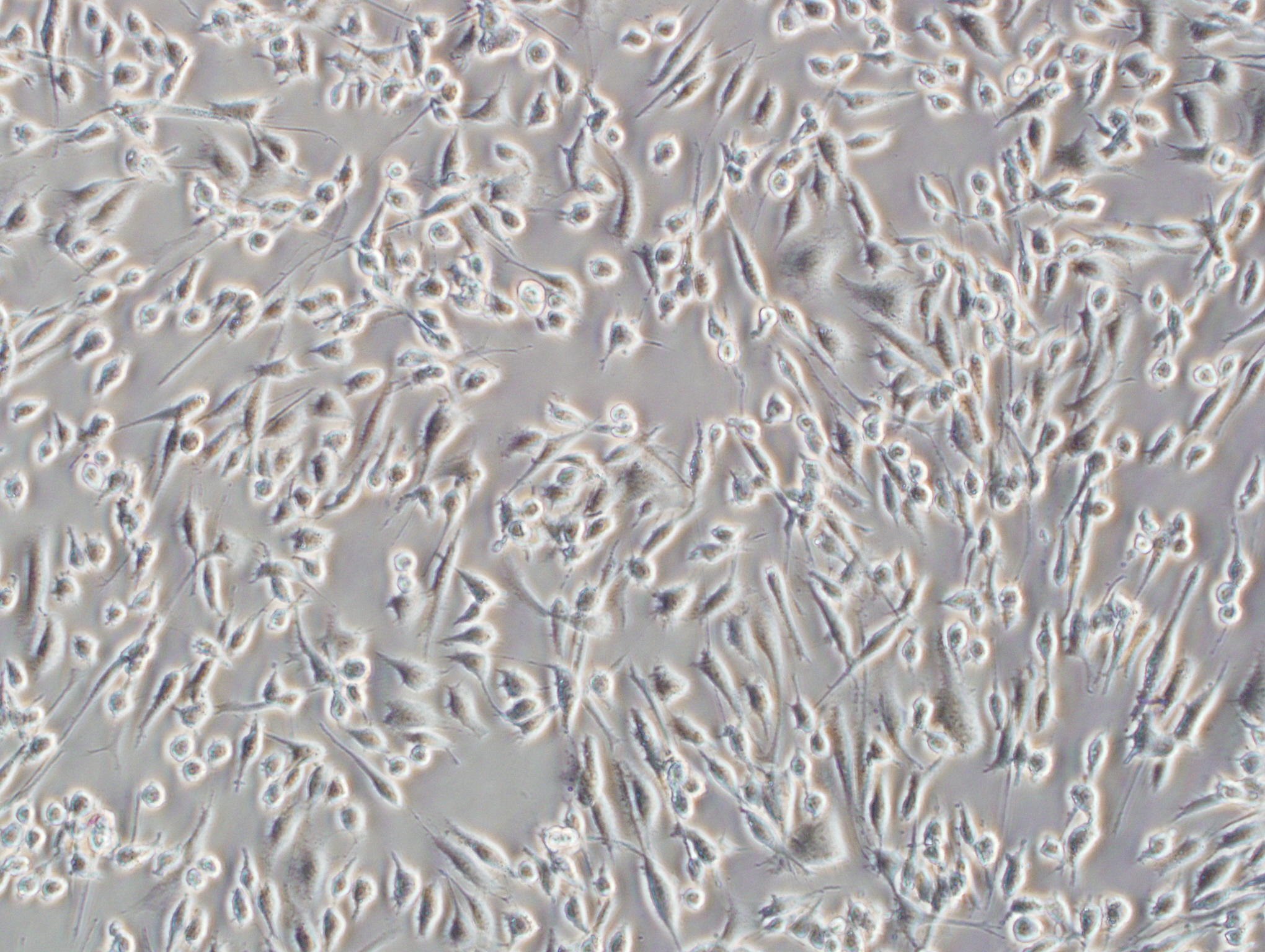

Supplement: Supplementary file 2 — Figure S2. The morphology of differential PC12 cells. (JPG 2204 kb) [file 12974_2018_1282_MOESM2_ESM.jpg]

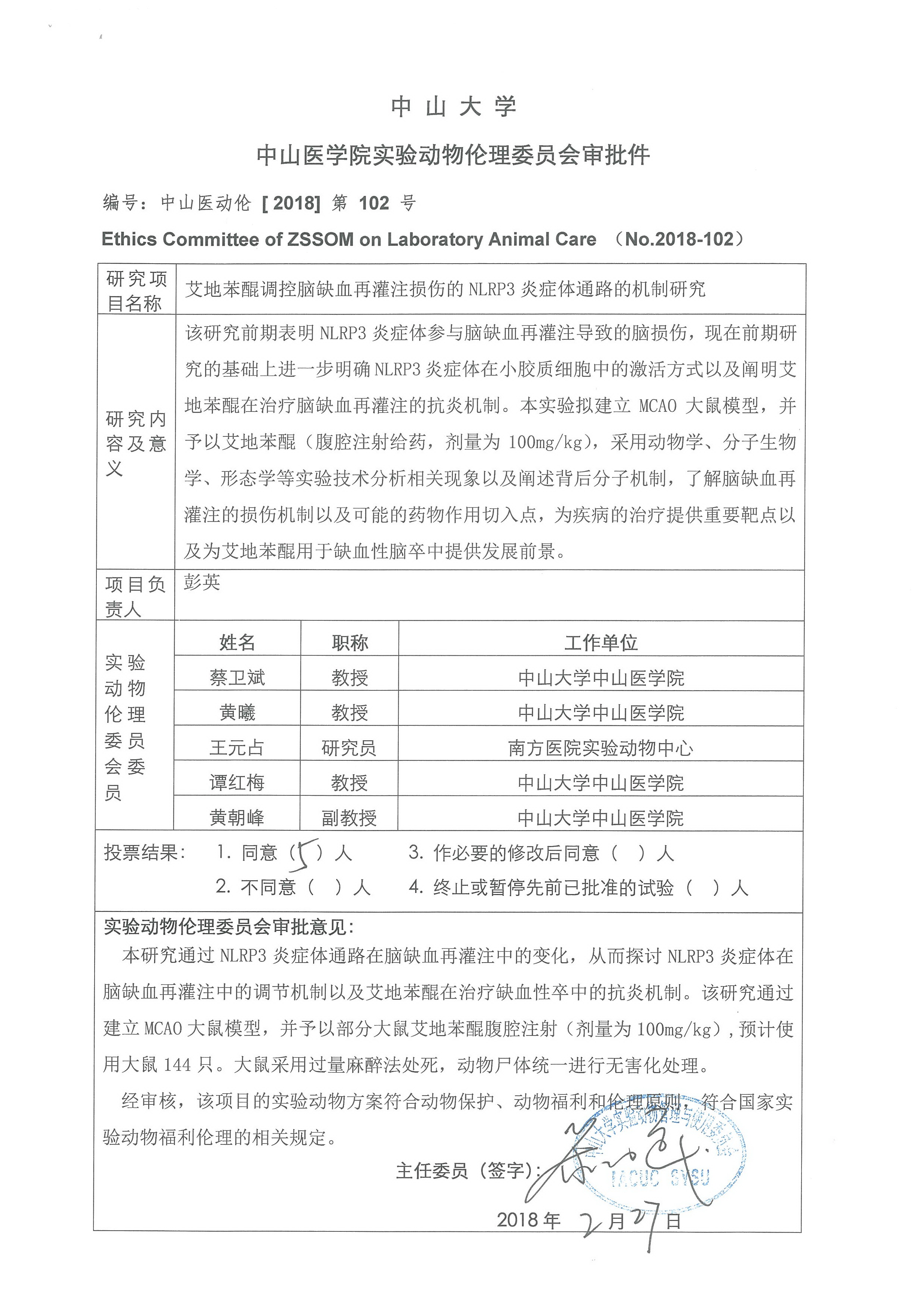

Supplement: Supplementary file 3 — Figure S3. Ethics approval of the animal usage. (JPG 1160 kb) [file 12974_2018_1282_MOESM3_ESM.jpg]
